# Effects of the competition schedule in major-competition years on performance in major-competition: Evidence from speed- and power-type track and field events

**DOI:** 10.1371/journal.pone.0351722

**Published:** 2026-07-16

**Authors:** Xiaolong Zhang, Xin Wei, Yao Yu, Tiantian Wang

**Affiliations:** School of Physical Education, Hubei University of Technology, Wuhan, China; ISSEP Kef: Universite de Jendouba Institut Superieur du Sport et de l’Education Physique du Kef, TUNISIA

## Abstract

**Objective:**

This study aimed to examine the association between competition schedules in major-competition years (Olympic or World Championships years, M-Years) and performance in major-competition (M-Performance) among athletes in speed- and power-type track and field events.

**Methods:**

This was a retrospective observational study involving athletes who competed in speed- and power-type track and field events at the World Athletics Championships or the Olympic Games between 1993 and 2019, yielding 14,584 valid observations. First, a two-step cluster analysis was conducted to identify typical M-Year competition schedules based on the annual total number of competitions and the proportions of performance states at each level. Next, baseline regression models were used to identify differences in M-Performance across competition-schedule types and to determine the “optimal competition schedule.” Finally, fixed-effects models were employed to explore the association between adjusting from a “suboptimal competition schedule” to the “optimal competition schedule” and M-Performance.

**Results:**

Athletes’ competition schedules in M-Years were classified into three types: “major-event–focused,” “competition-oriented,” and “competition-as-training.” Among these, athletes under the “competition-oriented” schedule exhibited significantly better M-Performance than those under the other two schedules. Further analysis showed that adjustment from a “competition-as-training” schedule to a “competition-oriented” schedule was associated with higher M-Performance, whereas adjustment from a “major-event–focused” schedule to a “competition-oriented” schedule showed no similar association.

**Conclusions:**

In M-Years, even the optimal competition schedule may not be suitable for all athletes, and its effectiveness may vary by athlete type. For “competition-as-training” athletes, adjusting toward a “competition-oriented” schedule may be associated with higher M-Performance. However, such adjustment may have limited benefits for “major-event–focused” athletes. To the best of our knowledge, this study is the first to conduct a large-scale empirical analysis of competition scheduling in major-competition years. The findings provide an empirical basis for future studies to further examine the causal relationship between competition-schedule adjustments and M-Performance, while also offering practical reference for athletes and coaching teams preparing for major events such as the Olympic Games and the World Athletics Championships.

## Introduction

Across most disciplines, the Olympic Games and the World Championships are regarded as the highest-caliber and most influential competitions. Athletes’ ability to display strong performance states and win in these events bears not only on personal honor and career development but can also, to some extent, affect national economic development [1] and national pride [2,3]. Consequently, determining how to help athletes reach their best performance states and contend for victory in such major events has remained one of the central research questions in high-performance sport.

According to existing research, scientifically planning the competition schedule in major-competition years (Olympic or World Championships years, M-Year) plays a critical role in athletes’ performance in major-competition (M-Performance) [4]. Prior studies have explored the impact of competition schedules on M-Performance from the perspective of participation quantity. For example, Born, Lomax [5] and Pearson, Spathis [6] argue that increasing competition frequency can help improve performance. However, Svendsen, Gleeson [7], in a study of elite cross-country skiers, found that frequent competition in M-Years increases illness risk among male athletes and ultimately impairs performance. With the deepening of research in recent years, the focus of analysis has expanded from “participation quantity” to “configuration of performance states during participation”, i.e., examining the relationship between competition schedules and M-Performance from the perspective of combinations of participations under different performance states. For instance, Bompa and Buzzichelli [4] further emphasize that the performance state at which athletes compete is more critical than competition count per se; competing too often in a peak state may degrade M-Performance. Accordingly, they recommend targeting only 2–3 competitions for peak-state performances via specific adjustment strategies, while approaching the remaining events with short tapers and a moderate performance state [4]. In contrast, Mujika, Villanueva [8] found among world-class swimmers that athletes who consistently entered all M-Year competitions in a high performance state achieved better M-Performance. This result indicates that the optimal competition schedule may vary significantly across different sports and athlete groups. Additionally, some coaches adopt “competition-as-training”—typically entering events with little specific preparation and a lower performance state—believing this enhances training adaptation and, in turn, improves M-Performance [4].

While the above studies demonstrate that M-Year competition scheduling materially affects M-Performance, they also reveal notable divergences in practical applications. On the one hand, there is no consensus on the mechanism by which participation quantity influences M-Performance. On the other hand, different performance states in competitions typically entail distinct pre-competition preparations and physiological load profiles. Treating all competitions within a season as homogeneous events and analyzing only based on participation quantity may obscure the key mechanism by which competition schedules—composed of combinations of different performance states—influence M-Performance.

Based on this, the present study draws on Bompa and Buzzichelli [3] by calculating the degree to which an athlete’s result in a given competition deviates from their annual best result, thereby attempting to transform the concept of “performance state” in training theory into an operational indicator applicable to large-scale competition data analysis. It should be emphasized that this classification based on relative performance does not constitute a direct measurement of athletes’ immediate physiological status or training preparation. Rather, it should be understood as an operationalized approximation under large-scale retrospective competition data. For this reason, the present study focuses more on the combinational characteristics of competitions performed under different performance states within a season and their empirical associations with M-Performance, rather than interpreting the performance-state indicator as a physiological diagnostic measure that is completely independent of the competition context.

Accordingly, this study uses track and field as a case, identifying typical competition-scheduling patterns within athletes’ M-Years through participation quantity and combinations of performance states, and empirically examining their associations with M-Performance. Because speed/strength athletes typically compete more frequently within a season than endurance athletes—yielding richer competition–performance time series for quantifying scheduling–outcome relationships—we excluded endurance-event samples. This research offers practical reference for athletes and coaching teams preparing for major events such as the Olympic Games and the World Athletics Championships.

## Methods

### Participants

Recognizing that the COVID-19 pandemic could distort athletes’ competition cycles and M-Performance, we excluded pandemic-affected years and therefore analyzed athletes who competed in speed/strength track-and-field events at the World Athletics Championships and the Olympic Games during 1993–2019, yielding 149,834 initial observations. After applying multiple exclusion criteria, 5,105 athletes remained, with a total of 14,584 observations meeting the study’s inclusion standards (see Table 1 for details). All data used in this study were obtained from publicly available resources provided by the official website of World Athletics (formerly the IAAF; https://www.iaaf.org/home). The datasets were accessed for research purposes between October 10, 2024 and 20 November 2024. Although some of the original records on the website contained information that could potentially identify individual athletes (e.g., names or nationalities), no identifiable or sensitive information was collected, extracted, or analyzed in this study. Only aggregated and non identifiable statistical data were used for research purposes. Consequently, no additional ethical review or informed consent was required, as all information originated from official, publicly accessible sources.

**Table 1:** The study sample information.

programme	Age (M/SD)	Gender (Number)
100 Metres	25.2(±4.02)	Male: 869 Female: 610
100 Metres-H	26.6(±3.68)	Male: 0 Female: 538
110 Metres-H	25.9(±3.43)	Male: 676 Female: 0
200 Metres	24.8(±3.75)	Male: 752 Female: 482
400 Metres	25.0(±3.60)	Male: 756 Female: 669
400 Metres-H	25.7(±3.58)	Male: 694 Female: 511
Discus Throw	28.7(±4.90)	Male: 594 Female: 341
Hammer Throw	27.2(±4.61)	Male: 621 Female: 498
High Jump	26.1(±3.91)	Male: 588 Female: 443
Javelin Throw	27.1(±4.35)	Male: 622 Female: 430
Long Jump	26.1(±3.91)	Male: 579 Female: 393
Pole Vault	26.1(±3.78)	Male: 491 Female: 408
Shot Put	27.3(±4.21)	Male: 575 Female: 449
Triple Jump	26.3(±3.98)	Male: 561 Female: 434
SUM	26.2(±4.13)	Male: 8378 Female: 6206

Note: 100 Metres-H = 100 Metres Hurdles; 110 Metres-H = 110 Metres Hurdles

400 Metres-H= 400 Metres Hurdles; M= mean; SD= standard deviation;

### Sample size estimation and justification

A power analysis for the R² test was conducted using Stata 18.0 to estimate the required sample size, with the significance level set at 0.05 and statistical power at 0.80. The minimum effect size standard for the model’s R² value was set at 0.0196, as there was a lack of precise information on the effect size for level of performance state or similar variables [9]. Based on a cluster analysis model, the regression model incorporated five covariates, specifically the proportions of four levels of performance states and the annual total number of competitions. The required sample size was calculated to be 648, a figure that was substantially lower than the actual sample size of 14584 utilized in this study.

### Study design

**Fig 1:**
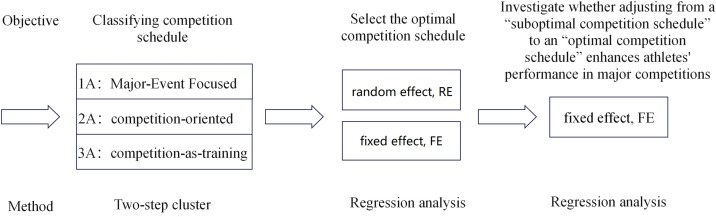
Flowchart of the study design. The first line of the figure presents the research objective. The second line provides specific research information and model selection. The third line gives a brief description of statistical methods.

To assess how M-Year competition schedules influence M-Performance, we first applied cluster analysis—using athletes’ number of competitions and the proportions of performance states at each tier—to identify several distinct types of competition schedules, and summarized their basic characteristics with descriptive statistics. Second, taking the type with the best M-Performance as the reference group, we used dummy-variable regression models to systematically examine the associations between different competition schedules and M-Performance, thereby identifying the “optimal competition schedule.” Finally, the study explored whether adjusting a “suboptimal competition schedule” to the “optimal competition schedule” is conducive to improving athletes’ M-Performance, and based on the results, revealed potential mechanisms to provide practical insights (Fig 1).

### Variables

#### Performance in major-competition (M-Performance).

Based on the research by Xin, Yaping [10], the performance state demonstrated by athletes in the World Athletics Championships and Olympic Athletics events is defined as their M-Performance, with the following calculation formulas: 1) For track events, M-Performance = (1 – Ratio _A_) × 100; For field events, M-Performance = (Ratio _A_ – 1) × 100. Ratio _A_ = the athlete’s best result in a specific World Athletics Championships or Olympic Athletics event divided by the athlete’s best result in the same year.

#### Performance states.

Based on the studies of Xin, Yaping [10], Bompa and Buzzichelli [4], we calculate the levels of performance states for track and field athletes based on their competition results. In sprint events: Level 1 of performance states = competition performance > −0.5; Level 2 of performance states = −0.5 ≥ competition performance > −1; Level 3 of performance states = −1 ≥ competition performance > −2; Level 4 of performance states = competition performance ≤ −2. In non-sprint events: Level 1 of performance states = competition performance > −1; Level 2 of performance states = −1 ≥ competition performance > −2; Level 3 of performance states = −2 ≥ competition performance > −3.5; Level 4 of performance states = competition performance ≤ −3.5 (Table 2). The formula for calculating competition performance is as follows: 1) In sprint events, competition performance = (1 – ratio _B_) × 100; 2) In field events, competition performance = (ratio _B_ – 1) × 100. Ratio _B_ = the athlete’s best result in a particular competition divided by the athlete’s best result in the same year.

**Table 2:** Levels of Performance States Classification Criteria.

Levels of Performance States	sprint events	non-sprint events
Level 1	per > −0.5	per > −1
Level 2	−0.5 ≥ per >−1	−1 ≥ per > −2
Level 3	−1 ≥ per > −2	−2 ≥ per > −3.5
Level 4	Per ≤−2	Per ≤−3.5

Note: per= Competition performance

#### Type of competition schedule.

In this study, the type of competition schedule is defined as a comprehensive characterization of the competitive behavior of speed- and power-type track and field athletes during major-competition years, and is the result of objective classification of athletes’ competitive behavior. Specifically, a two-step cluster analysis method was adopted, with the annual number of competitions and the proportion of each level of performance state as core indicators, to objectively classify athletes’ heterogeneous competitive behaviors, ultimately forming types of competition schedules with significant differentiation and stable internal homogeneity.

#### Adjustment of competition schedule.

In this study, adjustment of competition schedule is defined as the dynamic change in the type of competition schedule adopted by athletes across different years. Specifically, on the basis of determining the type of competition schedule for each athlete in each year, the adjustment direction of their competition schedule was identified by comparing the type of competition schedule of the same athlete in the previous competition year and the current year. For example, if an athlete was classified as Type A in the previous year and adjusted to Type B in the current year, their competition schedule adjustment was defined as “adjustment from Type A to Type B”.

### Data collection and processing

The sample in this study is derived from the statistical information published on the IAAF website (https://www.iaaf.org/home). The preprocessing of the sample data is as follows: 1) To ensure consistency in competition rules across age groups, only outdoor competition results were considered. 2) Results obtained under wind speed violations (≥2.0 m/s) and short-distance performances lacking electronic timing were excluded. 3) For athletes who competed in more than one event, the highest ranked event was selected. 4) For the same athlete in the same competition and event (e.g., preliminary, semifinal, and final results), only the best competition result was retained. 5) Only competition results from the years of the World Athletics Championships and the Olympic Games were retained. 6) Within the years of the above two major events, only competition results before the official start of these events were retained. 7) Athletes who did not participate in the World Championships or Olympic Games track and field events for various reasons were also excluded. 8) Samples lacking demographic information such as birth dates were excluded.

### Statistical

The dataset was imported into Stata 18.0 for statistical analysis. Because measurement units differ across events, athletes’ competition results were converted into standardized *z*-scores, and all other variables were standardized accordingly. First, a two-step cluster analysis was conducted to identify distinct sample categories, a method considered appropriate for large data sets [11]. To ensure the validity of the resulting clusters, the clustering solution was tested on a random subsample (50% of the dataset), and the level of agreement between the full-sample and subsample classifications was evaluated using the weighted Kappa statistic. The three-cluster solution demonstrated high consistency (Kappa = 0.85). Subsequently, a robustness test was conducted by adjusting the calculation parameters of the “performance state” variable to further evaluate the reliability of the results. Second, based on the clustering results, one type of competition schedule was chosen as the reference group to construct dummy-variable regression models, with M-Performance as the dependent variable and each type of competition schedule as the independent variable, to systematically test the relationships between different competition schedules and athletes’ M-Performance. Finally, an individual fixed-effects model was used to examine the specific impact of adjusting a “suboptimal competition schedule” to the “optimal competition schedule” on M-Performance. In this model, the dependent variable was M-Performance, and the independent variable was the method of competition schedule adjustment. It is worth noting that robustness tests were also conducted for the above two sets of regression models. Specifically, subsamples comprising 70% and 50% of the total sample were randomly selected for repeated testing. In addition, considering that both the dependent and independent variables were quantified using athletes’ annual best results, this may give rise to the issue of mathematical coupling. Therefore, we further constructed a null-hypothesis dataset for supplementary testing. Specifically, while keeping each athlete’s M-Performance unchanged, the corresponding performance states were randomly shuffled across athletes (For example, Athlete A’s M-Performance in a given year could be paired with Athlete B’s performance states in a given year). Subsequently, the two-step cluster analysis and dummy-variable regression analysis were repeated using the null-hypothesis dataset (To facilitate comparison with the original dataset, the number of clusters was fixed at three). If clustering and regression results similar to those of the original data could still be observed under this procedure, this would suggest that the original findings were mainly driven by mathematical coupling caused by the shared denominator. Conversely, if the original results could not be reproduced, this would indicate that the original findings reflected actual associations among the factors. The level of statistical significance was set at 0.05.

## Results

### Classification of competition schedules

This study examined the influence of M-Year competition schedules on the M-Performance of athletes in speed/strength track-and-field events. Variables such as the number of competition and the proportions of performance states at different levels were included to identify distinctive clusters, and all variables were standardized prior to analysis.

The results showed that: (1) Group 1A exhibited a low-frequency competition trend (mean frequency = 1.67), characterized by a dominant proportion of Level 1 performance states and relatively low proportions in other levels. (2) Group 2A showed a moderate competition frequency (mean frequency = 6.02), with Level 3 performance states being most prominent, moderate proportions of Levels 1 and 2, and a smaller share of Level 4. (3) Group 3A demonstrated a high-frequency competition trend (mean frequency = 8.87), with Level 4 performance states dominating and lower proportions of other levels. Subsequent ANOVA results indicated significant differences in annual best result and M-Performance among the three groups (*P* < 0.01). Although athletes in Group 3A exhibited higher overall annual best result, those in Group 2A achieved the best M-Performance (Table 3).

**Table 3:** Results of cluster analysis.

Cluster	1A	2A	3A
Level 1	1.52(±0.95) (96%)	**1.55**(±1.08) **(27%)**	1.27(±0.95) (16%)
Level 2	0.04(±0.19) (1%)	1.22(±1.21) (20%)	0.75(±0.97) (8%)
Level 3	0.03(±0.16) (1%)	**2.17**(±1.58) **(35%)**	1.73(±1.67) (17%)
Level 4	0.09(±0.30) (3%)	1.08(±0.90) (16%)	5.12(±2.71) (57%)
N-competitions	1.67(±1.23)	**6.02**(±2.66)	8.87(±3.89)
n	1015	5240	8329
**ANOVA** **(analysis of variance)**			
M-Performance (M/SD)	−2.97% (±2.76)	**−2.05%** (±2.93)	−3.32% (±3.88)
*P*	<0.01		
C-Level (M/SD)	0.560 (±0.70)	0.939 (±0.57)	**1.126** (±0.53)
*P*	< 0.01		

Note: Level 1 = Level 1 of performance states; Level 2 = Level 2 of performance states; Level 3 = Level 3 of performance states; Level 4 = Level 4 of performance states; C-Level = Competitive level; N- competitions = the number of competitions

Furthermore, to ensure the reliability and robustness of the clustering results, this study adjusted the calculation parameters of the performance state variable (unified to sprint-type calculation formula or non-sprint-type calculation formula). The results showed that the classification structure did not undergo substantial changes, indicating that the clustering results have good stability (see Table 4 for details).

**Table 4:** Robustness test results of cluster analysis.

Cluster	Sprint standard			Non-sprint standard		
1A	2A	3A	1A	2A	3A
Level 1	1.51 (96%)	1.58 (27%)	1.16 (13%)	2.01 (96%)	2.40 (39%)	1.45 (16%)
Level 2	0.02 (1%)	1.28 (22%)	0.36 (4%)	0.02 (1%)	1.97 (32%)	1.00 (11%)
Level 3	0.02 (1%)	1.81 (31%)	1.07 (12%)	0.02 (1%)	1.17 (19%)	2.54 (28%)
Level 4	0.05 (3%)	1.23 (21%)	6.22 (70%)	0.04 (2%)	0.55 (9%)	3.99 (44%)
Competitions	1.57	5.84	8.89	2.09	6.15	9.07
n	1009	6334	7241	1298	6816	6470

Note: Level 1 = Level 1 of performance states; Level 2 = Level 2 of performance states; Level 3 = Level 3 of performance states; Level 4 = Level 4 of performance states; Competitions = the number of competitions; Sprint standard = Unified to sprint-type calculation formula; Non-sprint standard = Unified to non-sprint-type calculation formula.

Finally, three null-hypothesis datasets were generated and subjected to two-step cluster analysis in sequence. The results showed that none of the three datasets reproduced the structural characteristics observed in the real dataset described above (S1 Table).

### Selection of the optimal competition schedule

We employed individual fixed-effects and random-effects models to construct dummy-variable regression models, with M-Performance as the dependent variable and competition schedule type as the independent variable (with Group 2A serving as the reference category). To enhance the robustness of the results, control variables including gender, competition age, event type, and competitive level were included in the model. The Hausman test (*P <* 0.01) supported the use of the individual fixed-effects model.

The results showed that in the random-effects model, athletes under the 1A and 3A competition schedules performed significantly worse in major competitions compared with those under the 2A schedule (*P <* 0.01). In contrast, in the fixed-effects model, only the result for Group 3A was significantly different (*P <* 0.01).

To ensure the reliability of the above results, robustness tests were conducted using random sampling. Subsamples comprising 70% and 50% of the total sample size were randomly selected to repeat the above regression analysis. The results showed that the effect sizes were consistent with the full-sample results in terms of coefficient direction and statistical significance, indicating that the results had good robustness (Table 5).

**Table 5:** Results of virtual variable regression analysis.

Variables	Full sample B(SE B		70% subsample B(SE B)		50% subsample B(SE B)	
RE	FE	RE	FE	RE	FE
**Independent Variables**						
1. 1A	−0.415^***^(0.110)	−0.064(0.159)	−0.546^***^(0.203)	−0.056(0.264)	−0.495^***^(0.238)	−0.108(0.365)
2. 3A	−0.614^***^(0.059)	−0.374^***^(0.074)	−0.830^***^(0.083)	−0.414^***^(0.106)	−0.814^***^(0.106)	−0.433^***^(0.148)
**Control Variables**						
1. Age	control	control	control	control	control	control
2. Gende	control	control	control	control	control	control
3. Sport discipline	control	control	control	control	control	control
4. C-Level	control	control	control	control	control	control
Ad *R*^2^	0.215	0.075				

Note: 70% subsample = Randomly sampled 70% subsample; 50% subsample = Randomly sampled 50% subsample; C-Level = Competitive level; RE = Random-effects models; FE = Fixed-effects models; ***p < 0.01; ** *p <* 0.05.

It is worth noting that the above tests were also conducted using the three null-hypothesis datasets. The results showed that, compared with the competition schedule associated with the best M-Performance in each null-hypothesis dataset, the other two competition schedules showed no significant differences in M-Performance (S2 Table). Combined with the clustering results from the null-hypothesis datasets, these findings suggest that the main results of this study were not mathematical pseudo-correlations automatically generated under a shared-denominator condition, but were more likely to reflect actual associations between athletes’ competition schedules and M-Performance.

Based on the above, we further posed the following research questions: 1) Whether adjusting athletes’ competition schedules from 3A to 2A, that is, from 3A in the previous year to 2A in the subsequent year, would be associated with higher M-Performance; and 2) whether adjusting athletes’ competition schedules from 1A to 2A, that is, from 1A in the previous year to 2A in the subsequent year, would show no association with higher M-Performance.

### Exploring the impact of adjusting to the “optimal competition schedule” on M-Performance

To address the above research questions, we employed an individual fixed-effects model for empirical analysis. The model was specified as follows: the dependent variable was athletes’ M-Performance, and the independent variable was the method of competition schedule adjustment (three-category variable: Category 1 = adjustment from 1A to 2A; Category 2 = adjustment from 3A to 2A; Category 3 = reference group, including other adjustment methods except the above two). The Hausman test result (p < 0.01) supported the use of the individual fixed-effects model.

The results indicated that when athletes’ competition schedules were adjusted from 1A to 2A, their M-Performance showed no significant difference compared to the reference group. In contrast, when athletes’ competition schedules were adjusted from 3A to 2A, their M-Performance was significantly improved compared to the reference group (*P <* 0.01). Furthermore, to ensure the reliability of the results, the study conducted robustness tests using random sampling. Subsamples of 70% and 50% of the total sample size were randomly selected to repeat the above regression analysis. The results showed that the effect coefficient of the group adjusted from 3A to 2A remained significantly positive at the 1% and 5% statistical levels, and there was no systematic deviation in the coefficient size, indicating that the results have good robustness (Table 6).

**Table 6:** Regression analysis of the impact of adjusting competition schedules on M-Performance.

Variables	B(SE B)		
	Full sample	70% subsample	50% subsample
**Independent Variables**			
1. 1A-2A	0.229(0.251)	0.142(0.306)	0.052(0.423)
2. 3A-2A	0.304^***^(0.094)	0.349^***^(0.115)	0.343^**^(0.163)
**Control Variables**			
1. Age	control	control	control
2. Gende	control	control	control
3. Sport discipline	control	control	control
4. C-Level	control	control	control
Ad *R*^2^	0.025		

Note: 1A-2A= When an athlete’s competition schedule was 1A in the previous year and 2A in the current year; 3A-2A= When an athlete’s competition schedule was 3A in the previous year and 2A in the current year; 70% subsample = Randomly sampled 70% subsample; 50% subsample = Randomly sampled 50% subsample; C-Level = Competitive level; ***p < 0.01; ** *p* < 0.05.

However, it should be noted that the R² of this model was only 0.025, indicating that approximately 97.5% of the variation in M-Performance could not be explained by competition schedule adjustment factors. Therefore, although this amount of explained variation may be considered practically meaningful in elite competitive sport, it should still be recognized that M-Performance is the result of multiple interacting factors. Compared with unobserved factors such as training load, illness status, and psychological preparation, competition schedule adjustment represents only one limited component.

## Discussion

### Three types of competition schedules

This study aimed to examine the association between competition schedules in M-Years and M-Performance among athletes in speed/strength track-and-field events. Based on a two-step cluster analysis incorporating athletes’ competition frequency and the proportion of each level of performance state, the sample was divided into three groups.

(1) Athletes in Group 1A had the lowest competition frequency among the three groups, averaging only 1.67 events per year. Most of their performances were classified as Level 1, representing a peak performance state, which typically requires a tapering phase of 8–14 days prior to the event [4]. Further examination of the raw data revealed that 1A athletes, aside from participating in major events (e.g., World Championships or other Olympic-level competitions), took part in only a few less important meets such as qualification events. Considering the training and preparation demands in elite sports, the remainder of their season was largely devoted to regular training. Early coaches and physiologists suggested that low competition frequency allows athletes to spend more time in training, thereby enhancing physical development [12] and improving training adaptation [13]. Based on these characteristics, this group was categorized as “major-event–focused”. The study found that athletes in this category demonstrated significantly lower competitive results and poorer M-Performance compared with those in Group 2A.
(2) Athletes in Group 2A exhibited a moderate competition frequency, participating in an average of 6.02 events per year. Across all competitions, they reached peak (Level 1) performance only in a few major events, while competing at a medium-to-high performance level (Levels 2–3) in most other meets. Typically, athletes can achieve a medium-to-high performance state after a short tapering period of 2–3 days [4], which theoretically supports the ability of Group 2A athletes to compete more frequently within a season. Further analysis of the raw data indicated that, aside from achieving peak form in a few key competitions, these athletes also participated extensively in many less significant events. Given that they typically performed in these competitions at medium-to-high levels, it can be inferred that Group 2A athletes often relied on brief 2–3 day tapers to prepare for the majority of non-major events. Bompa and Buzzichelli [4] explicitly supported this competition model, suggesting that throughout a season, athletes typically target peak performance in only about two events, while relying on short 2–3 day tapers for the rest. Based on these characteristics, this group was defined as “competition-oriented”. The study found that although “competition-oriented” athletes had lower average competitive results than those in Group 3A, their M-Performance in major competitions was significantly better than that of athletes in other categories.
(3) Athletes in Group 3A demonstrated a high competition frequency, averaging 8.87 events per year. Unlike the “competition-oriented” athletes, 3A athletes primarily competed at a low (Level 4) performance state, with medium-to-high (Levels 2–3) performance states occurring less frequently. Prior research indicates that competing at a low performance state generally requires no special pre-competition preparation, implying that these athletes remain in a phase of high-intensity training during competition without implementing tapering protocols [4]. This characteristic suggests that Group 3A athletes tend to treat competitions as high-intensity training sessions within their training cycle rather than as target events requiring specific preparation. Some professional coaches intentionally adopt this competition strategy, believing that frequent participation enhances athletes’ physical capabilities and ultimately helps them reach peak performance [4]. Therefore, this group was classified as “competition-as-training”. The study found that although these athletes had significantly higher average competitive results than those in other groups, their M-Performance was markedly lower than that of the “competition-oriented” athletes (see Table 7 for details).

**Table 7:** Typical Competition Structure for Speed/Power Track and Field Athletes.

Cluster	Competition Structure Characteristics	C-Level	M-Per
M-E-F	This group had the lowest competition frequency among the three types, averaging only 1.67 events per year, with most performances occurring in a Level 1 performance state.	Low	Medium
C-O	This group showed a moderate competition frequency among the three types, averaging 6.02 events per year. They reached a Level 1 performance state only in a few major competitions, while most other events were performed at Levels 2–3.	Medium	High
C-A-T	This group had the highest competition frequency among the three types, averaging 8.87 events per year. Most of their performances occurred at Level 4, while Levels 2–3 appeared relatively infrequently.	High	Medium

Note: M-E-F = Major-Event-Focused; C-O = Competition-Oriented; C-A-T = Competition-as-Training; C-Level = Competitive level; M-Per= M-Performance.

### Association between adjustment from “competition-as-training” to “competition-oriented” competition schedules and M-Performance

This study argues that adjusting athletes’ competition schedules from “competition-as-training” to “competition-oriented” is associated with higher M-Performance. Subsequent testing using an individual fixed-effects model supported this hypothesis. Specifically, an average 0.30% increase in athletes’ M-Performance was associated with this adjustment, which is close to the threshold of practically significant improvement (0.50%) as described by scholars such as Mujika and Padilla [14].

The application of a fixed-effects model requires that the core variables vary over time. Analysis of the raw data revealed that due to variations in the distribution of target competitions and training schedules across years [15], most athletes’ competition arrangements exhibited clear time-variation, meeting the core applicability conditions of the fixed-effects model. The Hausman test (*P <* 0.01) further confirmed that the use of a fixed-effects model was appropriate for the analysis.

From a training science perspective, we argue that the observed results are closely related to the reconstruction of athletes’ load tolerance. According to the nonlinear model proposed by Busso [16], the fatigue induced by a given training load depends on the intensity of the preceding training stimulus. The same exercise load induces greater fatigue when after by high-intensity training, but not when it follows a period of lower training load. This implies that the performance gains observed after tapering are, to some extent, the result of the reestablishment of athletes’ tolerance capacity [16,17]. Adjusting athletes’ competition schedules from “competition-as-training” to “competition-oriented” essentially represents a significant increase in the frequency of medium-to-high performance states, which indicates that athletes have eliminated part of the accumulated fatigue through short-term tapering. Under such conditions, identical competition stimuli induce less fatigue and of shorter duration, which clearly facilitates the execution of subsequent training plans and enhances eventual M-Performance. Conversely, when a larger number of competitions occur under low performance states, they generate greater and longer-lasting fatigue under heavy-load conditions [17], making it difficult for athletes to accumulate and convert training form into peak performance, thereby constraining their M-Performance. More critically, such conditions may even lead to overtraining syndrome (OTS) [18] or sports injuries [19], both of which can exert detrimental effects on athletes’ M-Performance [20,21].

Secondly, from a physiological perspective, we believe this is related to differences in the neuromuscular activation level and hormone content of athletes under different competition schedules. As mentioned earlier, athletes’ performance states generally show an upward trend after this adjustment. Studies have shown that athletes with better performance states have more sensitive muscle responses to neural drive, shorter neural delays during reaction time, and maintain an optimal testosterone-cortisol ratio [22,23]. At this time, athletes have higher movement power and lower fatigue levels. The above physiological changes lay the foundation for athletes to achieve higher performance in subsequent major competitions. In contrast, if the performance state is low before major competitions, the nervous system may enter a “hypoactive mode”. In this mode, even if training is intensified before major competitions, it is difficult for neural excitability to quickly return to the optimal state, ultimately exerting an adverse impact on major competitions.

Finally, from a psychological perspective, the enhancement of self‑efficacy and confidence may also contribute to the observed results. Previous studies have shown a positive relationship between competitive state and self-efficacy [24,25]. Higher self-efficacy is typically linked to more positive emotional states [26]. According to self-regulation theory, individuals use positive emotions as motivational energy to achieve their goals [27], which in turn fosters athletes’ willingness and determination to improve their performance [26]. Similarly, confidence has also been found to exhibit a stable positive correlation with competitive performance state [28]. Athletes with high levels of confidence are more capable of performing at their best under the pressure of major competitions and effectively coping with adverse situations during the event [29,30].

### Association between adjustment from “major-event–focused” to “competition-oriented” competition schedules and M-Performance

The study found that in the random-effects baseline regression model that did not fully control for individual heterogeneity, the M-Performance corresponding to the “competition-oriented” schedule was significantly better than that of the “major-event–focused” schedule. However, after introducing fixed effects, the difference in M-Performance between the two types of competition schedules weakened significantly, and we did not observe a significant improvement in M-Performance when adjusting from “major-event–focused” to “competition-oriented”, thus confirming Hypothesis 2.

First, we approach this from a statistical perspective. According to the research by D. Allison and Wu [31], replacing the standard error (SE = 0.110) in the random-effects model with that in the fixed-effects model did not change the results. This indicates that the difference between the two models mainly lies in the size of the coefficients. The most plausible explanation for this is that there are certain unobserved individual traits that can explain the positive impact we observed in the random-effects model, which are relatively stable over a specific period of time. When these variables are controlled for through the fixed-effects model, the impact disappears [31]. In fact, the random-effects model has an estimate of between-individual variation, and the additional information it contains gives it smaller sampling variability. However, this may come at a high cost. As mentioned earlier, this method cannot automatically eliminate the impact of time-invariant causes, thereby being affected by omitted variables [32]. This gives us sufficient reason to reject the use of the random-effects model and choose the fixed-effects model, even if its efficiency is affected [32].

Second, we approach this from the perspective of training practice. The average competitive level of athletes under the “major-event–focused” schedule is the lowest among the three types, which usually means they only have weak recovery ability, tactical stability, and competition adaptability, making it impossible for them to adopt or maintain the “competition-oriented” schedule for a long time. Therefore, in the random-effects baseline regression model, the descriptive advantage of the “competition-oriented” schedule reflects more individual differences among athletes rather than the direct causal effect of the competition schedule itself. In other words, the “competition-oriented” schedule is more likely to be a result formed by high-level athletes during their competitive development, rather than a competitive strategy that can be independently applied and universally replicated.

This study still has several limitations. First, the analysis was primarily based on athletes in speed- and power-oriented track and field events. Future research should broaden the range of sport disciplines included in the sample to establish the external validity of the current findings. Second, athletes’ performance states were calculated based on competition results; however, competition results may be influenced by external factors such as tactical arrangements, the level of opponents, and weather conditions. Although these nonsystematic errors tend to exert relatively minor effects on the overall results in large-sample analyses, future studies could incorporate physiological monitoring indicators or subjective fatigue ratings as complementary measures to evaluate and calibrate athletes’ performance states, thereby further enhancing the scientific rigor of the indicator. Finally, the retrospective design of this study cannot fully capture the specific intentions of coaches and athletes when formulating competition schedules. For example, some athletes may have competed less frequently not because their coaches actively chose a more conservative competition strategy, but because of unrecorded minor injuries, insufficient recovery, or restrictions related to qualification procedures. Similarly, some athletes may have competed frequently and completed competitions in lower performance states due to factors such as commercial participation obligations or team selection strategies. Therefore, the findings of this study should still be understood as associational evidence based on real-world competition data, rather than as causal training prescriptions that can be directly translated into active intervention strategies. Future research should employ prospective cohort studies, quasi-experimental designs, or randomized controlled trials, while incorporating multidimensional indicators such as training load, injury history, recovery status, sleep quality, psychological preparation, and pre-competition tapering processes, to further examine the causal effects of competition schedule adjustment on athletes’ M-Performance.

## Conclusion

The present study found that competition scheduling in M-Years was significantly associated with M-Performance among speed/power track and field athletes. Among the three typical competition schedules, athletes under the “competition-oriented” schedule achieved the best M-Performance. Further analyses showed that adjustment from a “competition-as-training” schedule to a “competition-oriented” schedule was associated with higher M-Performance, whereas adjustment from a “major-event–focused” schedule to a “competition-oriented” schedule showed no similar association. These findings suggest that athletes and coaching teams should tailor season planning to individual characteristics, emphasizing cyclical regulation and strategic allocation rather than relying on a single “optimal” competition schedule. Future research could employ prospective designs in multi-sport samples, particularly randomized controlled trials or quasi-experimental studies, to further examine the causal effects of competition schedule adjustment on M-Performance.

## Supporting information

S1 Table Clustering Analysis Results for the Zero-Hypothesis Dataset. The table contains the results of three randomizations.
(DOCX)

S2 Table Results of Regression Analysis on the Zero-Hypothesis Dataset. The table contains the results of three randomizations.
(DOCX)
