# Supplementary material for: Effects of the competition schedule in major-competition years on performance in major-competition: Evidence from speed- and power-type track and field events
Source: PLoS One. 2026 Jul 16;21(7):e0351722. doi: 10.1371/journal.pone.0351722 (PMC13374908; doi:10.1371/journal.pone.0351722)
Supplement: S1 Table — The table contains the results of three randomizations. (DOCX) [file pone.0351722.s001.docx]

**S1 Table. Clustering Analysis Results for the Zero-Hypothesis Dataset.** The table contains the results of three randomizations.

| first randomization | | | |
| --- | --- | --- | --- |
| Cluster | 1A | 2A | 3A |
| One | **3.57(±1.65) (46%)** | 1.01(±1.10) (15%) | 0.87(±0.77) (15%) |
| Two | 0.82(±1.06) (8%) | 0.56(±0.74) (7%) | 1.10(±1.04) (21%) |
| Three | 1.80(±1.66) (18%) | 0.98(±1.00) (12%) | 1.96(±1.42) (38%) |
| Four | 2.33(±1.93) (25%) | 3.96(±2.41) (65%) | 1.45(±1.19) (24%) |
| Competitions | 8.68(±4.72) | 6.13(±3.96) | 5.46(±2.67) |
| n | 4952 | 5505 | 4127 |
| **ANOVA (analysis of variance)** | | | |
| M-Performance (M/SD) | **-2.31% (±2.80)** | -3.51% (±3.68) | -2.36% (±3.86) |
| *P* | ＜0.01 |  |  |
| C-Level (M/SD) | 1.034 (±0.56) | **1.085 (±0.55)** | 0.937 (±0.62) |
| *P* | ＜0.01 | | |
| second randomization | | | |
| Cluster | 1A | 2A | 3A |
| One | **2.38(±1.69) (52%)** | 1.16(±1.37) (21%) | 1.83(±1.75) (18%) |
| Two | 0.14(±0.48) (2%) | 1.78(±1.92) (35%) | 0.85(±0.96) (9%) |
| Three | 1.51(±1.39) (28%) | 0.46(±0.98) (7%) | 3.49(±1.58) (38%) |
| Four | 1.11(±0.98) (17%) | 1.82 (±0.96) (35%) | 3.12(±2.27) (34%) |
| Competitions | 5.00(±2.94) | 4.97(±2.75) | 9.00(±4.41) |
| n | 3753 | 2748 | 8083 |
| **ANOVA (analysis of variance)** | | | |
| M-Performance (M/SD) | **-2.23% (±3.15)** | -2.34% (±3.02) | -3.09% (±3.68) |
| *P* | ＜0.01 |  |  |
| C-Level (M/SD) | 0.910 (±0.65) | 0.996 (±0.58) | **1.084 (±0.53)** |
| *P* | ＜0.01 | | |
| third randomization | | | |
| Cluster | 1A | 2A | 3A |
| One | 1.57(±1.47) (34%) | 2.42(±1.87) (26%) | 1.39(±1.20) (21%) |
| Two | 0.05(±0.22) (1%) | 1.53(±1.02) (20%) | 0.47(±0.87) (7%) |
| Three | 0.51(±0.96) (8%) | 1.37(±1.42) (15%) | **2.74(±1.34) (45%)** |
| Four | 2.95(±2.32) (55%) | 3.32(±2.64) (38%) | 1.80(±1.52) (25%) |
| Competitions | 5.19(±2.87) | 8.71 (±4.40) | 6.53 (±3.07) |
| n | 4221 | 6185 | 4178 |
| **ANOVA (analysis of variance)** | | | |
| M-Performance (M/SD) | -2.98% (±3.38) | -2.84% (±3.41) | **-2.35% (±3.65)** |
| *P* | ＜0.01 |  |  |
| C-Level (M/SD) | 0.944 (±0.63) | **1.096 (±0.53)** | 0.989 (±0.58) |
| *P* | ＜0.01 | | |
| Note：One = Level 1 of performance states; Two = Level 2 of performance states; Three = Level 3 of performance states; Four= Level 4 of performance states; C-Level = Competitive level, Calculated based on the highest annual performance; Competitions= the number of competitions | | | |
