# Supplementary material for: Effects of the competition schedule in major-competition years on performance in major-competition: Evidence from speed- and power-type track and field events
Source: PLoS One. 2026 Jul 16;21(7):e0351722. doi: 10.1371/journal.pone.0351722 (PMC13374908; doi:10.1371/journal.pone.0351722)
Supplement: S2 Table — The table contains the results of three randomizations. (DOCX) [file pone.0351722.s002.docx]

**S2 Table.** **Results of Regression Analysis on the Zero-Hypothesis Dataset.** The table contains the results of three randomizations.

| first randomization | | | |
| --- | --- | --- | --- |
| Variables | B（SE B） | | |
|  | RE | FE | |
| **Independent Variables** |  | | |
| 1. 2A | 0.030（0.063） | -0.007（0.073） | |
| 2. 3A | -0.013（0.063） | -0.011（0.075） | |
| **Control Variables** |  | | |
| 1. Age | control | control | |
| 2. Gende | control | control | |
| 3. Sport discipline | control | control | |
| 4. C-Level | control | control | |
| Ad $R^{2}$ | 0.219 | 0.073 | |
| second randomization | | | |
| Variables | B（SE B） | | |
|  | RE | FE | |
| **Independent Variables** |  | | |
| 1. 2A | -0.095（0.077） | -0.086（0.090） | |
| 2. 3A | 0.020（0.063） | -0.037（0.075） | |
| **Control Variables** |  | | |
| 1. Age | control | control | |
| 2. Gende | control | control | |
| 3. Sport discipline | control | control | |
| 4. C-Level | control | control | |
| Ad $R^{2}$ | 0.219 | 0.073 | |
| third randomization | | | |
| Variables | B（SE B） | | |
|  | RE | FE | |
| **Independent Variables** |  | | |
| 1. 1A | 0.141（0.069） | 0.123（0.080） | |
| 2. 2A | 0.047（0.061） | 0.054（0.071） | |
| **Control Variables** |  | | |
| 1. Age | control | control | |
| 2. Gende | control | control | |
| 3. Sport discipline | control | control | |
| 4. C-Level | control | control | |
| Ad $R^{2}$ | 0.219 | 0.072 | |
| Note：C-Level = Competitive level, Calculated based on the highest annual performance; ****p* ＜0.01；** *p* ＜0.05. | | |  |
